# Tolerance of Rare-Earth Elements in Extremophile Fungus *Umbelopsis isabellina* from Polar Loparite Ore Tailings in Northwestern Russia

**DOI:** 10.3390/jof9050506

**Published:** 2023-04-23

**Authors:** Oleg I. Shumilov, Elena A. Kasatkina, Irina Y. Kirtsideli, Dmitry V. Makarov

**Affiliations:** 1 Institute of North Industrial Ecology Problems, Kola Science Centre, Russian Academy of Sciences, 184209 Apatity, Russia; o.shumilov@ksc.ru (O.I.S.); d.makarov@ksc.ru (D.V.M.); 2 Komarov Botanical Institute, Russian Academy of Sciences, 197376 Saint Petersburg, Russia; microfungi@mail.ru

**Keywords:** *Umbelopsis isabellina*, extremophile microfungi, Arctic, loparite ore tailings, rare earth tolerance

## Abstract

In this study, extremophile fungal species isolated from pure loparite-containing sands and their tolerance/resistance to the lanthanides Ce and Nd were investigated. The loparite-containing sands were collected at the tailing dumps of an enterprise developing a unique polar deposit of niobium, tantalum and rare-earth elements (REEs) of the cerium group: the Lovozersky Mining and Processing Plant (MPP), located in the center of the Kola Peninsula (northwestern Russia). From the 15 fungal species found at the site, one of the most dominant isolates was identified by molecular analysis as the zygomycete fungus *Umbelopsis isabellina* (GenBank accession no. OQ165236). Fungal tolerance/resistance was evaluated using different concentrations of CeCl_3_ and NdCl_3_. *Umbelopsis isabellina* exhibited a higher degree of tolerance/resistance to cerium and neodymium than did the other dominant isolates (*Aspergillus niveoglaucus*, *Geomyces vinaceus* and *Penicillium simplicissimum*). The fungus began to be inhibited only after being exposed to 100 mg L^−1^ of NdCl_3_. The toxic effects of Ce were not observed in fungus growth until it was subjected to 500 mg∙L^−1^ of CeCl_3_. Moreover, only *U. isabellina* started to grow after extreme treatment with 1000 mg∙L^−1^ of CeCl_3_ one month after inoculation. This work indicates, for the first time, the potential of *Umbelopsis isabellina* to remove REEs from the loparite ore tailings, making it a suitable candidate for the development of bioleaching methods.

## 1. Introduction

The Arctic and Antarctic polar regions are considered to be stressed habitats. However, despite their cold climates, many microfungal species have been found in these regions that can grow and survive under extreme conditions: low and high temperatures, lack of water, low nutrient availability, UV radiation, etc. [1,2,3,4,5,6,7,8,9,10,11,12,13,14,15,16,17,18,19,20,21,22]. Cold-tolerant microfungi known as psychrophiles have been found in the polar soils, water, snow, ice and rocks [2,7,8,10,11,12,13,14,15,16,17,18,19,20,21]. For instance, mycelial fungi have been isolated from cryopegs (mineralized headwater lenses below the Arctic massive ground ice bodies) in the Kolyma lowland of northeastern Russia [2]. These microorganisms have gained wide attention due to their ability to produce cold-adapted enzymes and bioactive natural compounds with an exceptional application potential in the pharmaceutical field, including antioxidants, antibiotics, anticancer and antidiabetic drugs, and sunscreens [6,8,10,20,21]. In particular, some Arctic and Antarctic aquatic fungi have been proven to be a source of α-glycosidase inhibitors, which are regarded as effective antidiabetic drugs [8,21]. Some representatives of this group, the endolithic microfungi, can also colonize bare rocks in hot and cold deserts [4,10,12,18,19]. Endolithic communities play an important role in global biogeochemical processes; they participate in the bioweathering and transformation of rocks and minerals, as they are able to form young soils and mycorrhiza for pioneer plant species [4,23]. These abilities mean that endoliths are capable of acting as pioneering microorganisms that can modify uninhabited environments [4,19,23]. Thus, these microfungi may represent models for astrobiological studies [10,12,17,19]. Microfungi from these and other extreme environments (extremophiles) exhibit special morphological and physiological adaptations to avoid different stresses [10,19]. The study of these extremophile fungi may provide tools for understanding the molecular processes that allow them to maintain metabolic activity under conditions that are lethal for most organisms. Recently, there has been growing interest in studies of extremophile microfungal species, with the aim of exploring and developing new and more nature-like biotechnological applications [10,13,14,21].

In addition, some microfungi may be able to colonize technogenic landscapes created by anthropogenic activities, such as those polluted with oil and mining products, heavy metals, toxic chemicals, municipal solid waste, etc. [9,10,15,16,24,25,26,27]. Microfungal strains isolated from these environments are able to tolerate and detoxify high concentrations of heavy metals [10,27], e.g., *Penicillium simplicissimum* and *Aspergillus foetidus* show higher rates of growth in the presence of heavy metals [28]. These strains may also be considered as potential sources of important antioxidants and bioactive compounds of importance in medicine and the chemical industry [10]. Because of their abilities, some microfungi are now used in the bioremediation of polluted areas around mines and industries, and as well as in bioleaching of metals from minerals, ore and mine tailings, industrial waste and electronic-product waste [27,29,30].

In recent decades, there has been an increasing trend in the research of microfungal species that interact with rare-earth elements [29,31,32,33,34,35,36]. However, current knowledge concerning REE tolerance (toxicity) in microfungi is still quite limited [33,34,36]. Rare-earths are naturally distributed in the environment and are now increasingly used in agricultural and high-tech-manufacturing industries [37]. Despite this, the toxicity of REEs to human health—and to the environment—is not yet fully understood [37].

**Figure 1:**
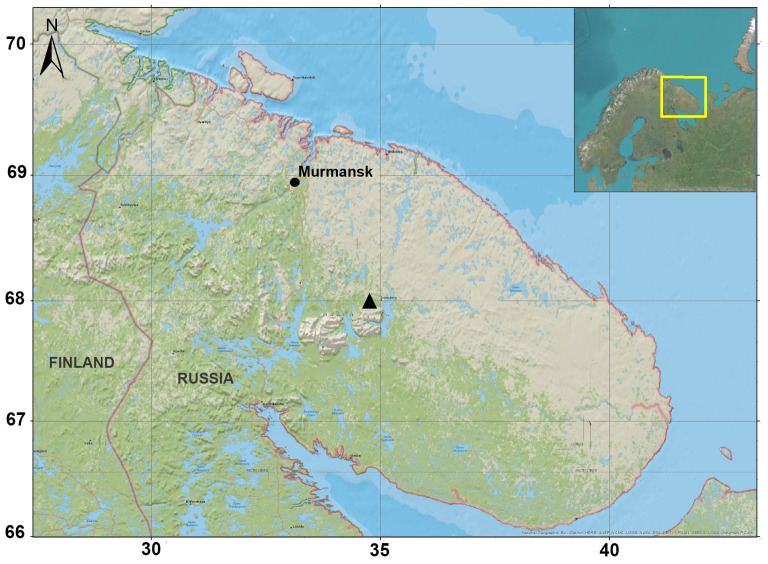
Map showing the sampling location at Lovozersky MPP (black triangle).

The Kola Peninsula is one of the most industrially developed and urbanized areas in the Arctic zone of Russia. The region is characterized by a unique combination of severe climatic conditions and intensive industries, including aluminum and copper–nickel smelters and mining and processing industries, among others. On the territory of the Kola Peninsula there are about 60 large deposits of mineral raw materials, the most valuable of which are apatite–nepheline, copper–nickel, iron and rare-earth ores [38]. One preliminary estimate of the expected value of existing REE reserves on the Kola Peninsula gave a figure of about USD 700 billion [39]. The only active deposit of loparite ores is located in the center of the peninsula (Figure 1). Mining and processing of loparite (a unique source of REEs) are carried out at Lovozersky Mining and Processing Plant (MPP). Loparite concentrate obtained at the site is used for further production of tantalum, niobium, titanium and the cerium group of rare-earth elements [38]. Proportions of REEs in loparite are as follows: 57.5% Ce, 28% La, 8.8% Nd and 3.8% Pr [40]. The loparite ore tailings contain a sufficient amount of REEs for them to be considered as “technogenic REE deposits” [38]. Although the traditional method of chemical leaching of REEs from mine tailings has been well explored, the bioleaching process for REE extraction from minerals already mined has not received sufficient attention to date [29].

The aim of this study was, firstly, to identify with molecular analysis the fungal strains isolated from the loparite ore concentration tailings and, secondly, to evaluate their tolerance/resistance to two lanthanides (Ce and Nd) using different concentrations of cerium chloride and neodymium chloride.

## 2. Materials and Methods

### 2.1. Sampling

**Figure 2:**
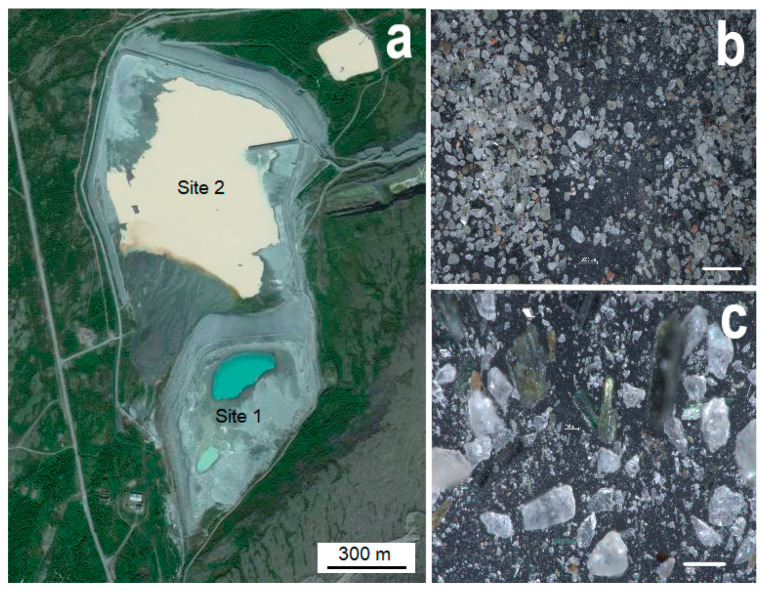
Map of the tailing dump with sites S1 and S2 (**a**) and samples of the loparite sand (**b**,**c**). Bars are 2000 µm (**b**) and 300 µm (**c**), respectively.

Almost the entire territory of the Kola Peninsula lies within the Arctic Circle. The climate is characterized as a subarctic, with an average annual temperature of about −1.5 °C. Samples for the mycological research were taken from two sites (S1 and S2) of the loparite tailing dump of the Lovozersky MPP near the settlement of Revda, Murmansk Oblast (67.9° N, 34.6° E; Figure 1 and Figure 2a). Waste accumulated for many years (1951–1985) at the first site S1; from 1985 to the present, waste accumulation has continued at the second site S2 (Figure 2a).

The tailings are fine-grained sands of dark gray color with the mineral particles of 0.01–0.5 mm size, in which individual grains of alkaline aluminosilicates, rare grains of pinkish-red eudialyte and dark green prisms of aegirine are clearly visible (Figure 2b,c). Mineralogical analysis has revealed that the tailings of loparite ores are mainly composed of nepheline, aegirine and feldspars [38]. Loparite, sodalite and apatite have also been found in impurity amounts [38]. It has also been previously established that the tailings are characterized by high amounts of Sr (1289 mg/kg) and Ce (1031 mg/kg), and lower amounts of Zn (240 mg/kg), La (202 mg/kg), Nd (121 mg/kg) and Pr (39 mg/kg) [41]. Details of the analytical procedures were previously reported by Krasavtseva et al. in 2021 [38]. In their study, the qualitative composition of the samples was controlled by powder X-ray diffraction on a DRON-2.0 X-ray diffractometer using CuKα radiation; the content analysis was performed using an ELAN 9000 DRC-e inductively coupled plasma mass spectrometer (by Perkin Elmer, Waltham, MA, USA) at the Kola Geological Information and Laboratory Center, Kola Science Centre (KSC RAS) [38]. The choice of Ce and Nd for analysis was due to the relatively high content of these elements in the loparite ore tailings.

### 2.2. Morphological and Molecular Identification

Fungi were isolated by several dilutions followed by sowing sand suspension on a Sabouraud dextrose agar (SDA) [42,43]. The strains were cultivated in the dark at 5 °C and 20 °C; then, isolated species were identified based on the observation of cultural and morphological characteristics such as the color of the colony and sporulation [44]. The morphological analysis was carried out with the light microscope Carl Zeiss AxioImager A1 (Germany). The broad-spectrum antibiotic chloramphenicol (100 mg/L) was added to the culture medium to suppress the growth of bacteria. Pure monospore cultures were obtained by sieving a suspension of spores and mycelium fragments prepared in a solution of 0.2% agar and 0.05% Tween 80 on a medium of Czapek (CZ) and SDA [45]. The initial identification of fungal species was carried out based on their macro- and micromorphological features in accordance with standard procedure after their isolation in a pure culture [44]. The names and positions of fungal taxa were unified using the Index Fungorum database [46]. Colonies were counted after 10–15 days (20 °C) and 30 days (5 °C) of cultivation. Data on fungal densities are expressed in colony-forming units (CFU) per gram of absolutely dry material (CFU/g). The contribution of each species to the structure of the microbiota was estimated as a relative abundance (number of isolates of a particular species in the sample/total number of all isolates in the sample, expressed as a percentage).

Some isolates were also identified with molecular methods. The cultures employed for molecular studies were cultivated on Czapek’s agar (CZ) at 20 °C for 14 days. The DNA was extracted using the Diamond DNA Plant kit (ABT, Russia, Barnaul) according to the supplier’s instructions. The internal transcribed spacer rDNA region (ITS1-5.8S-ITS2) was amplified using the PCR-primers ITS1 (5′-TCC-GTA-GGT-GAA-CCT-TGC-GG-3′) and ITS4 (5′-TCC-TCC-GCT-TAT-TGA-TAT-GC-3′) [47]. After amplification, the samples were detected by an electrophoretic method in 1.5% agarose gel with GelRed. The obtained DNA fragments were sequenced using the equipment of the Core Centrum Genomic Technologies, Proteomics and Cell Biology, All-Russia Research Institute for Agricultural Microbiology (ARRIAM).

Phylogenetic analysis was performed using the maximum likelihood method based on the Tamura–Nei model [48]. Sequences were inspected and assembled using the MEGA7 software package [49]. Newly generated sequences were compared with data sets from GenBank (National Center for Biotechnology Information, NCBI) [50] using the Basic Local Alignment Search Tool (BLAST) [51]. Additionally, the following criteria [52] were applied to interpret the sequences from the GenBank database: for query coverage and sequence identities of ≥98%, the genus and species were accepted; for query coverage and sequence identities between 95% and 97%, only the genus was accepted. The phenological tree was constructed by applying a neighbor-join (NJ) algorithm to a matrix of pairwise distances estimated using the maximum composite likelihood (MCL) approach. The quality of the branching patterns for NJ was assessed by bootstrap resampling of the data sets with 1000 replications. The genome sequence of the isolated and identified strain LVZ4 was deposited at the NCBI database under the accession number shown in Table 1.

### 2.3. REE Tolerance Testing

Fungal isolates and the control strain were tested for their tolerance to the rare-earth elements Ce and Nd. The microfungus *Sydowia polyspora* (Bref. & Tavel) E. Mull, which is a typical subarctic representative [53], was chosen as a control strain. The study of the morphology and growth rate of strains depending on the concentration of REE salts was carried out on a solid nutrient medium. Metal stock solutions were prepared by dissolving their chloride salts: cerium chloride heptahydrate CeCl_3_∙7H_2_O (>99.5%) and neodymium chloride hexahydrate NdCl_3_∙6H_2_O (>99.5%). The stock solutions of individual elements were added separately to the SDA medium. A series of solutions with different concentrations (0–10,000 mg/L) were prepared by diluting the stock Ce and Nd solutions with sterilized water. A 1 mL amount of each of these series of solutions was mixed with 9 mL of SDA media and then poured into plates. The isolates and control strain were then inoculated on SDA with three replicates. The control strain was inoculated on SDA without solutions. The strains were cultured on Petri plates (diameter 90 mm) in the dark at 20 °C for 14 days. Colony diameters were measured daily in two directions across the colonies. The measurements were made in triplicate. At the end of the experiment, changes in macro- and micromorphology were recorded. The growth rate (mm per day) was calculated according to a regression equation. The inhibition rates IR (%) of metals against the microfungi were calculated according to the following formula [35]:

IR (%) = (D_c_ − D_t_)/(D_c_ − D_0_) × 100%, (1)

where D_c_ is the diameter of control (cm), D_t_ is the diameter of REE treatment (cm), and D_0_ is the diameter of inoculum (cm).

### 2.4. Statistical Analysis

Experiments on fungal tolerance were performed in triplicate, and the results are expressed as means. The growth rate of each colony was calculated through linear regression. For cluster analysis, the results of the REE-growth inhibition for all strains were combined and compared. The data were examined using the weighted pair group method with arithmetic mean (WPGMA). Spearman correlation-based distance was used to combine clusters and draw a dendrogram. The dendrogram contained clusters, which grouped the strains with similar tolerance characteristics.

The statistical analysis was performed with the MATLAB statistical software package.

## 3. Results

### 3.1. Fungal Identification and Phylogenetic Analysis

In total, 15 species of microscopic fungi were morphologically identified from the two sites of the Lovozersky MPP’s loparite ore concentration tailings. The isolates represented ten genera and two sterile unidentified morphotypes. The average density of microfungal isolates in the tailings was very low. The preserved site of the tailings dump S1 contained only 8 ± 2 CFU/g. However, the density of microfungal isolates at the exploited site S2 was higher (12 ± 4 CFU/g). The dominant species were *Geomyces vinaceus* (20% of relative abundance on each of the sites, S1 and S2), *Penicillium simplicissimum* (18% and 19%, respectively) and strain LVZ4 (14% and 5%, respectively). The relative abundance of other isolates did not exceed ten percent. The significantly higher percentage of strain LVZ4 at the preserved site S1 was a noteworthy finding. A similar result was obtained for *Aspergillus niveoglaucus* (9% at site S1, compared with 3% at site S2).

**Figure 3:**
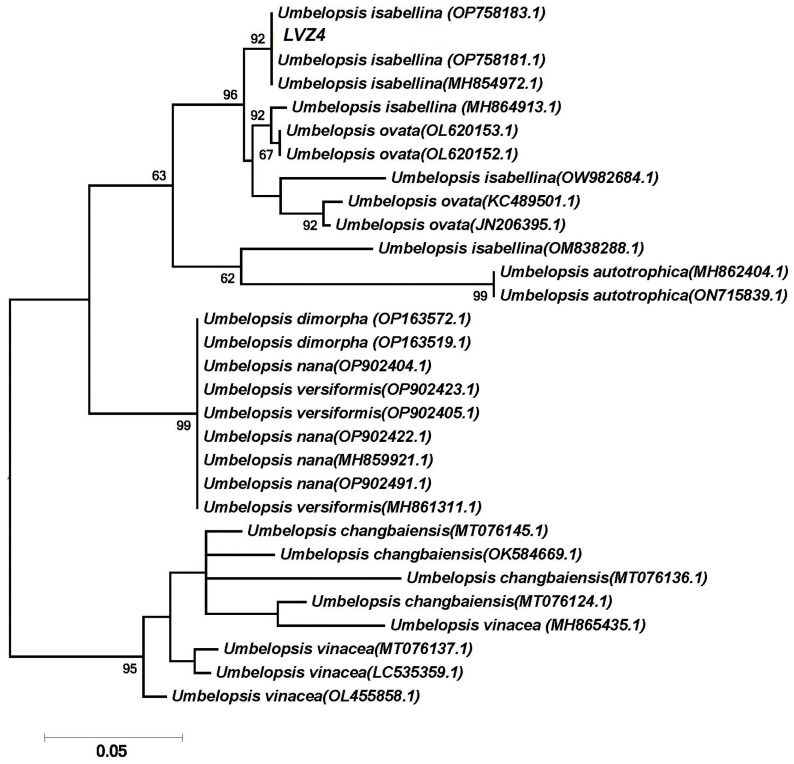
Maximum-likelihood phylogenetic tree of *Umbelopsis isabellina* LVZ4 and the representative sequences of the GenBank database. Numbers at the nodes indicate the bootstrap values (>60%) from 1000 replications.

The isolate LVZ4 was not initially identified morphologically and first appeared under the name *Sterile white*. A BLAST search of ITS rDNA revealed that the isolate LVZ4 matched *Umbelopsis isabellina* (GenBank accession no. MH854972.1) with a similarity value of 99%. Phylogenetic analysis of the ITS sequences suggested that the LVZ4 strain was placed within the same clade with *U. isabellina* (Figure 3). The obtained sequence has been submitted to the GenBank and has been assigned the accession number OQ165236 (see also Table 1).

### 3.2. Tolerance of Umbelopsis Isabellina to Different Concentrations of Ce and Nd

**Figure 4:**
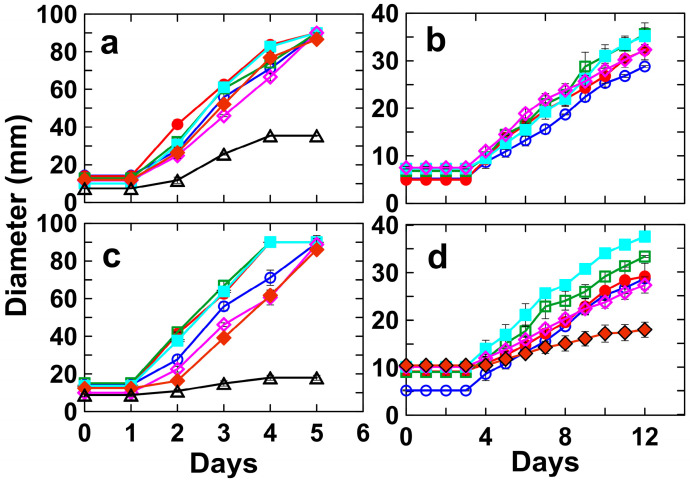
Colony expansion rates of *Umbelopsis isabellina* (**a**,**c**) and *Sydowia polyspora* (**b**,**d**) on the SDA media containing 0 mg/L (blue, empty circle), 12.5 mg/L (red, filled circle), 25 mg/L (green, empty square), 50 mg/L (cyan, filled square), 100 mg/L (purple, empty diamond), 250 mg/L (brown, filled diamond), 500 mg/L (black, empty triangle) of CeCl_3_ (**a**,**b**) and NdCl_3_ (**c**,**d**) over incubation at 20 °C in the dark. Data are averages of at least three replicates with error bars showing the standard error of the mean.

The degree of REE tolerance exhibited by *U. isabellina* (isolated) and *S. polyspora* (non-isolated) was determined based on the growth of fungi in the presence of Ce and Nd. Colony diameters were measured daily at different CeCl_3_ and NdCl_3_ concentrations of (0—control, 12.5, 25, 50, 100, 250, 500 and 1000 mg/L). These two strains exhibited very different responses to REEs (Figure 4 and Table 1). For instance, *S. polyspora* grew on REE-free SDA plates at an average rate of 2.41 ± 0.12 mm/day, which was not significantly influenced (*p* < 0.05) by the presence of 12.5, 25, 50 or 100 mg/L of either CeCl_3_ or NdCl_3_. However, at 250 mg/L of CeCl_3_, the growth of *S. polyspora* completely stopped, and its growth was only 0.81 ± 0.06 mm/day at 250 mg/L of dCl_3_ (see Figure 4d and Table 1). Conversely, *U. isabellina* continued to grow at 500 mg/L of both CeCl_3_ and NdCl_3_ (Figure 4a,c and Table 1).

**Figure 5:**
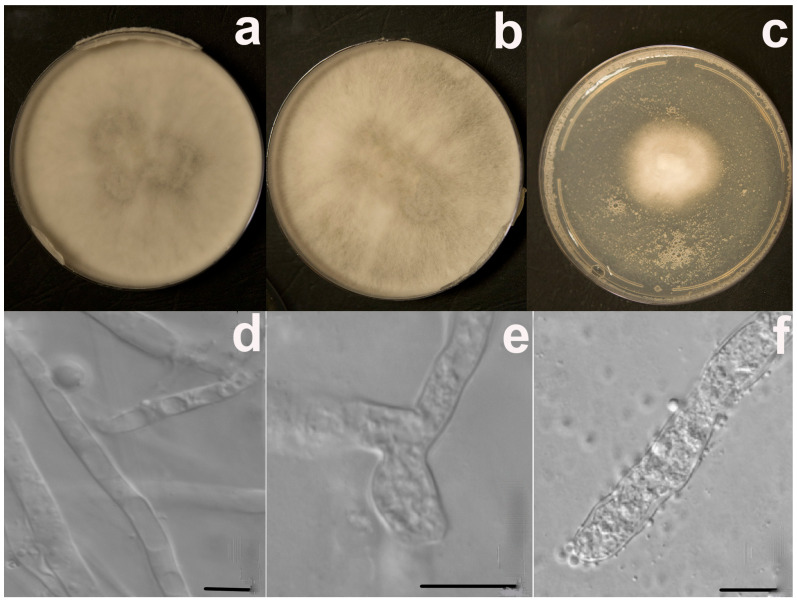
Effects of different concentrations of CeCl_3_ on the morphology of the extremophile fungal isolate *Umbelopsis isabellina* after 7-day incubation at 20 °C in the dark cultured in the Sabouraud dextrose agar media without CeCl_3_ (**a**,**d**), with 250 mg/L of CeCl_3_ (**b**,**e**) and with 500 mg/L of CeCl_3_ (**c**,**f**). Scale bars 10 µm, magnification 1000× (**d**–**f**).

The colony of *U. isabellina* treated with 250 mg/L of CeCl_3_ completely covered the plate on the seventh day, and this was similar to the control plate (Figure 5a,b). Over the same period, there were practically no changes in the morphology of colonies; those that were initially white, with a velvety aspect, became light gray with a cream shade after seven days at 20 °C in an SDA (Figure 5a,b). Colony development at the highest REE concentrations caused stress and changes in morphology. The colony under treatment with 500 mg/L of CeCl_3_ was white and much smaller (by almost three times) than that of the control (Figure 5c).

**Figure 6:**
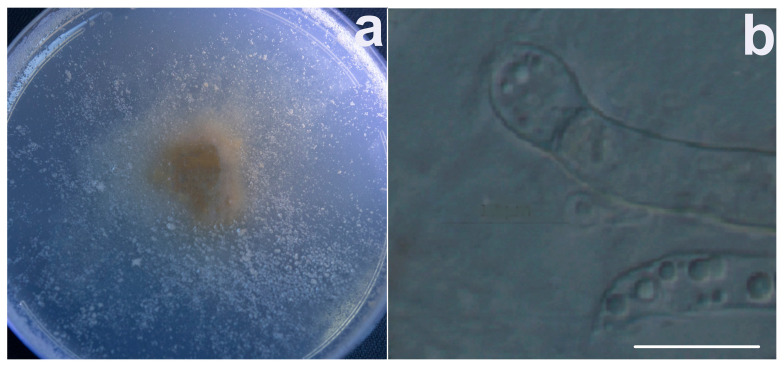
Inhibitory effects of 1000 mg/L of CeCl_3_ on the morphology of *Umbelopsis isabellina* after two months of incubation at 20 °C in the dark cultured in the Sabouraud dextrose agar media: (**a**) colony growth and (**b**) micromorphological characteristics; scale bar 10 µm, magnification 1000×.

Contrarily, micromorphological adaptations in response to stress caused by an increased REE content were already observed under the treatment with 250 mg/L of CeCl_3_ (Figure 5e). In fact, in the culture growing on a control medium (without REE’s salts), the presence of the sterile mycelium was noted (Figure 5d). Mycelium, which was initially sterile and homogeneous, became heterogeneous with swellings and the beginning of the formation of rounded structures inside them at 250 mg/L of CeCl_3_ (Figure 5e). Growth of *U. isabellina* at 500 mg/L of CeCl_3_ was accompanied by the production of mainly spherical chlamydospores up to 3–3.5 µm in diameter (Figure 5f). Interestingly, *U. isabellina* under treatment with 1000 mg/L CeCl_3_ started to grow slowly a month after inoculation in SDA (Figure 6). The color of the colony changed from an initial white-beige to beige with a pinkish tinge (Figure 6a). The aerial mycelium was practically absent for two months; the formation of rounded cells of various sizes was also noted (Figure 6b).

**Figure 7:**
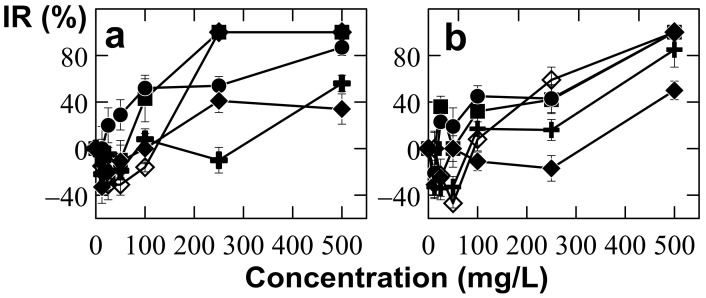
Inhibitory effects of CeCl_3_ (**a**) and NdCl_3_ (**b**) on the diameter growth of the selected fungi: *Aspergillus niveoglaucus* (filled circle), *Geomyces vinaceus* (filled square), *Penicillium simplicissimum* (filled diamond), *Sydowia polyspora* (empty diamond), and *Umbelopsis isabellina* (cross).

A comparative analysis of the inhibiting effects of Ce and Nd on *U. isabellina* and three other dominant isolates (*Aspergillus niveoglaucus*, *Geomyces vinaceus* and *Penicillium simplicissimum*) is shown in Figure 7. In this figure, IR variations of the control strain *S. polyspora* at different concentrations of Ce and Nd are also presented. The growth rate of *S. polyspora* and *G. vinaceus* was completely inhibited at 250 mg/L CeCl_3_, and their inhibition rates were close to 50% at 250 mg/L of NdCl_3_ (Figure 7). Similarly, the IR value for *A. niveoglaucus* was close to 50% at 250 mg/L of CeCl_3_ and NdCl_3_ (Figure 7). Contrarily, the inhibition rates for *U. isabellina* were negative (−10%) and slightly positive (16%) under treatments with 250 mg/L of CeCl_3_ and NdCl_3_, respectively (see Table 1). Furthermore, the inhibition rates for *P. simplicissimum* were 41% and −17% at 250 mg/L of CeCl_3_ and NdCl_3_, respectively (Figure 7). Negative IR values indicated the stimulation of fungal growth at small (12.5–50 mg/L) rare-earth concentrations. Finally, only *U. isabellina* and *P. simplicissimum* experienced growth stimulation under treatments with 250 mg/L of CeCl_3_ and NdCl_3_, respectively (Figure 7 and Table 1).

**Figure 8:**
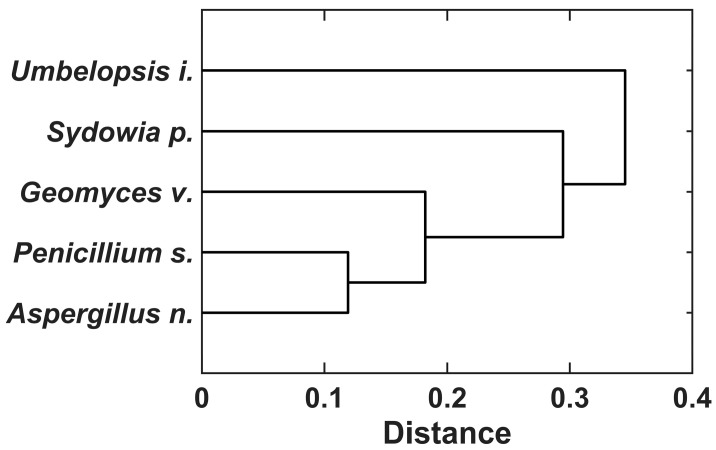
Dendrogram based on the Spearman distance and the WPGMA clustering method showing the different growth patterns of the selected microfungi.

The dendrogram of fungal clustering obtained by the weighted linkage method, which took into account the results of Ce and Nd responses, demonstrated two major clusters (Figure 8). The first cluster included the isolate *U. isabellina* alone, thereby confirming its considerable difference from other strains. The second major cluster was subdivided into two subgroups. The first subgroup contained the control strain *S. polyspora*, while the second subgroup combined the isolates *A. niveoglaucus*, *G. vinaceus* and *P. simplicissimum* (Figure 8). All the results suggested that *U. isabellina* was capable of surviving at high concentrations of Ce and Nd, and exhibited a high degree of resistance to the toxic effects of these REEs.

## 4. Discussion

Fungi of 15 species were isolated from pure loparite-containing sands, which were collected at the tailing dump of an enterprise developing a unique polar REE deposit on the Kola Peninsula. The average density of microfungi was extremely low, and it decreased with the age of the ore tailings. Thus, the amount of micromycetes was 50% higher (12 ± 4 CFU/g) in sands from the exploited site S2 than in sands from the preserved site S1 (8 ± 2 CFU/g), which were stored about 40 years ago. This finding contrasts with the results of other studies, which reported that average microfungal density increases with the age of mining waste [9,15]. For instance, in 10-year-old nepheline sands from the tailing dump of an apatite-nepheline mining enterprise located on the Kola Peninsula, the abundance of micromycetes has increased 24 times compared to freshly deposited sands, reaching a level of 300 CFU/g [15]. The extremely low fungal density found in our study can probably be explained by the special extreme conditions in the loparite ore tailings, i.e., low temperatures and nutrient deficiency. Taking into account these conditions, the loparite-containing sands from the tailing damp of Lovozersky MPP should be considered as an extreme environment. Contrarily, the number of fungi found in the dumps of coal mines in Spitsbergen, where micromycetes were provided with a sufficient level of organic carbon, exceeded the values obtained in our case by more than an order of magnitude [9]. It is noteworthy that the abundance of the dominant isolate *U. isabellina* was significantly higher (by three times) in the samples taken from S1 as compared with S2 in contrast with other dominant species, although the total number of isolates decreased. We may say, therefore, that the fungus *U. isabellina* has adapted to survive and grow in this extreme environment. Our findings are similar to those of other studies which reported that *U. isabellina* is widely distributed in soils of cold and moderately cold regions and is both psychrophilic and oligotrophic [4,6,7,15,16]. For instance, strains identified as *U. isabellina* have been successfully isolated from fine granitic sediments of the Damma glacier in the central Swiss Alps [4]. This fungus has also exhibited a high ability to weather powdered granite material in batch experiments [4]. Additionally, *U. isabellina* has also been found in natural and polluted soils of the Kola Peninsula [16], as well as in bottom sediments of the Barents and Kara seas [7]. Moreover, *U. isabellina* is an oleaginous fungus which can produce high amounts of lipids under specific growth conditions [54]. These lipids can be used as precursors for the synthesis of lipid-based biofuels [54]. Recently, the ability of *U. isabellina* to reduce the toxicity of environmental pollutants (heavy metals, phenolic xenobiotics, etc.) has also been reported [55,56]. However, there have been no reports to date about the interaction of *U. isabellina* with REEs.

In this study, the tolerance/resistance of *U. isabellina* to Ce and Nd was evaluated. The choice of cerium and neodymium for study purposes was due to the relatively significant content of these elements in the waste of loparite production compared to other REEs: Ce (1031 mg/kg) and Nd (121 mg/kg) [41]. Moreover, these elements are extremely important in a wide range of industries, including the manufacturing of modern technologies. Cerium and neodymium are important in electronics and are used in the production of plasma screens, lasers and smartphones, each of which contains 50 mg of Nd [37]. Nd-Fe-B magnets are the strongest permanent magnets known [37]. The recovery of REEs from loparite waste helps sustainable development through the circular economy and by creating more nature-like technologies. Tolerance studies can also help to assess the potential of *U. isabellina* as a bioleaching tool. According to our results, the growth of *U. isabellina* was stimulated when CeCl_3_ and NdCl_3_ concentrations ranged from 12.5 to 50 mg/L, whereas it was only partly inhibited when the NdCl_3_ concentration was greater than 100 mg/L and the CeCl_3_ concentration was greater than 500 mg/L (Figure 6 and Table 1). The growth of fungus under 100~250 mg/L CeCl_3_ was slightly stimulated or close to zero (Table 1). A comparative analysis with other isolates showed that only *U. isabellina* continued to grow at the highest Ce and Nd concentrations. It is especially worth noting that *U. isabellina* was capable of surviving at 1000 mg/L of CeCl_3_ and started its growth a month after inoculation in SDA. Cluster analysis also confirmed a considerable difference of *U. isabellina* from other isolates. To date, species described in studies as REE-resistant have been mainly from the *Aspergillus* and *Penicillium* genera [31,32,33,34,36,57]. Our findings indicate that *U. isabellina* exhibited greater physiological responses and higher tolerance mechanisms in response to Ce and Nd stress than did *A. niveoglaucus* and *P. simplicissimum*. Overall, our study demonstrated that *U. isabellina* was capable of surviving at high Ce and Nd concentrations and of exhibiting high resistance to the toxic effects of these REEs.

The findings of the present study suggest that high concentrations of Ce and Nd can stimulate the metabolism in *U. isabellina* to prioritize the production of resistant structures instead of reproductive ones. Indeed, the growth of *U. isabellina* at 500 mg/L CeCl_3_ was accompanied by production of chlamydospores after seven days of inoculation in SDA. A similar morphology with the presence of lipid-rich chlamydospores has been reported during the growth of *U. isabellina* on glucose [54]. The chlamydospore is a thick-walled asexual spore which is well adapted to maintaining fungus viability in extreme conditions [58].

The varied responses of in *U. isabellina* to different REE concentrations might be due to one or more types of tolerance/resistance strategies. The most important of these mechanisms are the following: chemical transformation, or dissolving of components by the organic acids secreted by fungi; biosorption, or metabolism-independent binding to the cell surface; and bioaccumulation, or energy-dependent flux into the cell [30]. The ability of *U. isabellina* to dissolve the granite material due to the release of citrate and malate has been reported by Brunner et al. [4]. A recent study by Janicki et al. [56] demonstrated that *U. isabellina* was capable of removing heavy metals from aqueous solutions, mainly by biosorption.

The higher tolerance of *U. isabellina* to Ce and Nd compared to other isolates indicates a possible bioleaching potential of the fungus.

## 5. Conclusions

To the best our knowledge, this is the first report on the REE-bioleaching potential of the fungus *U. isabellina*, which we isolated from Arctic loparite ore concentration tailings. *U. isabellina* is an oleaginous extremophilic fungus which can grow and survive in extreme environments. These characteristics make *U. isabellina* an attractive resource for more nature-like and eco-friendly industrial processes. Our study demonstrated, for the first time, the high degree of tolerance of *U. isabellina* to the lanthanides Ce and Nd. The degree of tolerance/resistance of *U. isabellina* to Ce and Nd was estimated based on the measurements of growth inhibition in REE-treated cultures. At levels of up to 500 mg/L, neither CeCl_3_ nor NdCl_3_ completely inhibited the *U. isabellina* growth. Moreover, only *U. isabellina* under treatment with 1000 mg/L of CeCl_3_ started its growth a month after inoculation. Because *U. isabellina* displayed a higher resistance to cerium and neodymium, this fungus should be considered as a model for the bioleaching of REEs. Future research should be conducted to improve the REE-bioleaching potential of *U. isabellina*.

## Figures and Tables

**Table 1:** The growth and inhibition rate (IR) shown by analyzed strains at different levels of cerium and neodymium chloride concentrations.

Species	Collection Number	Bank Accession Number	Concentration (mg/L)	Growth Rate (mm/day)	IR (%)
*Umbelopsis isabellina* (Oudem.) W. Gams	Control				
	LVZ4	OQ165236	0	16.44 ± 1.87	0
			Ce		
			12.5	17.46 ± 1.83	−22
			25	17.10 ± 1.75	−5
			50	18.51 ± 2.14	−19
			100	16.50 ± 2.05	8
			250	16.95 ± 2.03	−10
			500	7.43 ± 1.55	56
			Nd		
			12.5	20.27 ± 2.86	−33
			25	20.16 ± 2.78	−34
			50	20.27 ± 3.03	−33
			100	16.27 ± 2.13	17
			250	15.39 ± 2.67	16
			500	2.22 ± 0.32	85
*Sydowia polyspora* (Bref. & Travel) E.Mull (control strain)	Control				
			0	2.41 ± 0.12	0
			Ce		
			12.5	2.74 ± 0.11	−15
			25	2.97 ± 0.11	−31
			50	2.88 ± 0.16	−31
			100	2.54 ± 0.12	−16
			250	0	100
			500	0	100
			Nd		
			12.5	2.41 ± 0.12	0
			25	2.49 ± 0.12	−23
			50	2.87 ± 0.14	−47
			100	1.74 ± 0.09	8
			250	0.81 ± 0.06	59
			500	0	100

## Data Availability

Not applicable.
